# Clinical Features and Genetic Analysis of 48 Patients with Chronic Granulomatous Disease in a Single Center Study from Shanghai, China (2005–2015): New Studies and a Literature Review

**DOI:** 10.1155/2017/8745254

**Published:** 2017-01-30

**Authors:** Jing Wu, Wei-Fan Wang, Yi-Dan Zhang, Tong-Xin Chen

**Affiliations:** ^1^Department of Allergy and Immunology, Shanghai Children's Medical Center, Shanghai Jiao Tong University School of Medicine, Shanghai 200127, China; ^2^Division of Immunology, Institute of Pediatric Translational Medicine, Shanghai Jiao Tong University School of Medicine, Shanghai 200127, China; ^3^Department of Internal Medicine, The Affiliated Hospital to Changchun University of Chinese Medicine, Changchun 130021, China

## Abstract

Chronic Granulomatous Disease (CGD) is a rare inherited primary immunodeficiency, which is characterized by recurrent infections due to defective phagocyte NADPH oxidase enzyme. Nowadays, little is known about Chinese CGD patients. Here we report 48 CGD patients in our single center study, which is the largest cohort study from Mainland China. The ratio of male to female was 11 : 1. The mean onset age was 0.29 years old, and 52% patients had an onset within the 1st month of life. The mean diagnosis age was 2.24 years old. 11 patients (23%) had died with an average age of 2.91 years old. 13 patients (28%) had positive family histories. The most prevalent infectious sites were the lungs (77%), followed by gastrointestinal tract (54%), lymph nodes (50%), and skin (46%). In addition, septicopyemia, thrush, and hepatosplenomegaly were also commonly observed, accounting for 23%, 23%, and 40% of the cases. Lesions due to BCG vaccination occurred in more than half of the patients. X-linked CGD due to* CYBB* gene mutations accounted for 75% of the cases, and 11 of them were novel mutations. Autosomal recessive inheritance accounted for 6% patients, including 1 patient with* CYBA,* 1 with* NCF1*, and 1 with* NCF2* gene mutations.

## 1. Introduction

Chronic Granulomatous Disease (CGD; OMIM number 306400), which was firstly described in 1957 [1, 2], is a rare inherited primary immunodeficiency (PID). CGD is caused by defect in one of the subunits of nicotinamide dinucleotide phosphate (NADPH) oxidase, resulting in failure of phagocyte to generate superoxide. In neutrophil phagosome, superoxide combines with hydrogen ions to generate hydrogen peroxide, which is important for the intracellular killing of microorganisms [3]. As a result, CGD patients usually suffer from severely recurrent, often life-threatening bacterial and fungal infections [4–6].

NADPH oxidase is a transmembrane enzyme complex which is comprised of gp91^phox^, p22^phox^, p47^phox^, p67^phox^, p40^phox^, and Rac1/2 GTP binding protein subunits [7, 8]. It has been reported that CGD could be caused by defects in any of the components of this oxidase [3]. The most common reason leading to CGD is the defect on gp91^phox^, which is encoded by* CYBB* gene, localized on chromosome Xp21.1. Mutations on* CYBB* gene lead to X-linked recessive CGD (XL-CGD), which accounts for about 70–75% of CGD patients. Autosomal recessive CGD (AR-CGD) is mostly caused by defects in 1 of the 3 components of NADPH oxidase: p47^phox^ (encoded by* NCF1* gene, localized on chromosome 7q11.23), p22^phox^ (encoded by* CYBA* gene, localized on chromosome 16: 16q24), and p67^phox^ (encoded by* NCF2* gene, localized on chromosome 1q25), accounting for about 20%, 5%, and 5% CGD patients, respectively. Defects on p40^phox^, which is encoded by* NCF4* gene, localized on chromosome 22q13.1, could also lead to AR-CGD [4, 6, 9]. Besides, a defect on Rac2 (p.D57N) was also reported to be associated with human phagocytic immunodeficiency [9].

The incidence of CGD is approximately 1 case in 200,000 newborns in the United States [10] and Europe [11]. In Asia, the largest cohort study was from Japan, in which 229 patients were enrolled and the incidence was estimated to be about 1 out of 220,000 birth [12, 13]. The clinical course varied in CGD patients and most of them had an onset within the first year of life. Infections in skin, lung, gastrointestinal tract, lymph nodes, and liver were often observed in CGD patients. Granulomata, which has potential threat to cause ureteral, urinary bladder, esophagus or stomach obstruction, was also reported in CGD patients [4, 11, 14]. Many researches have been conducted in CGD patients worldwide over the last decades, however, little is known about Chinese CGD patients. In fact, although some CGD patients have been reported from Mainland China, most of the researches mainly focused on the molecular defects and BCG infections in the patients, providing little clinical information [15–17]. There are no established CGD registry in China and thus make a comprehensive and systematic analysis of CGD on Chinese individuals difficult. Further studies are still needed to clarify the clinical characteristics, complications, epidemiology, genetic information, and outcomes on Chinese CGD population.

Shanghai Children's Medical Center (SCMC) is a pediatric tertiary referral teaching hospital in Shanghai, China, which is one of the biggest PID centers in Mainland China. Here, we report 48 CGD patients who were diagnosed in our PID center from 2005 to 2015, which is the largest CGD cohort study from Mainland China. Furthermore, 157 CGD patients reported previously are reviewed in this paper, which aim to summarize and provide further insight into the characterization of CGD in China.

## 2. Patients and Methods

### 2.1. Patients

During 2005–2015, a total of 48 patients who were diagnosed with CGD were enrolled in this study. The diagnosis of CGD was based on the diagnostic criteria for primary immunodeficiency by Pan-American Group for Immunodeficiency (PAGID) and European Society for Immunodeficiencies [18]. The classification of CGD was based on the recommendation of the International Union of Immunological Societies Expert Committee for primary immunodeficiency (2015) [19].

This study was approved by the Ethical Committee of Shanghai Children's Medical Center, and written informed consent was obtained from all the participants.

CGD patients who were reported in Mainland China previously in English and Chinese literatures were reviewed in this paper. Most of the patients were reported from Children's Hospital of Chongqing Medical University (Center 2), Children's Hospital of Fudan University (Center 3), and Beijing Children's Hospital (Center 4).

### 2.2. Data Collection

The patients' detailed clinical and laboratory data were retrospectively collected from the patients' medical records, including clinical manifestations, age at onset and diagnosis, parental consanguinity, family history and frequency of infections, lesions induced by inoculation with BCG, laboratory tests, treatments, and outcomes.

The onset age was defined as the age when obvious infections initially occurred in the patients. The diagnosis age was taken as the age when CGD-related gene mutations were identified or low respiratory burst activation in neutrophils was found in the patients. Bacillus Calmette-Guerin (BCG) infections were divided into 3 categories according to the previous study: (1) regional infection, which included abscess or ulcer formation at the site of vaccination or ipsilateral axillary, supraclavicular, and cervical lymph node enlargement; (2) distant infection, which involved at least 1 distant site beyond regional ipsilateral infection, such as osteomyelitis, distant skin, and pulmonary infections; (3) disseminated infection, which involved more than 1 infection as mentioned in the distant infection [33].

### 2.3. DHR Flow Cytometric Assay

DHR flow cytometry assays were performed as previously described. In brief, 50 *μ*l whole peripheral blood was used in this assay. Firstly, the erythrocytes were lysed using 1x lysis buffer, then the leukocytes were loaded with DHR at 37°C for 5 minutes in the presence of catalase. After that, the leukocytes were activated with PMA for 15 minutes and the leukocytes were immediately analyzed by flow cytometry. Data were analyzed using Cell Quest software (BD FACSCalibur TM Flow Cytometer).

### 2.4. Genetic Analysis

Genomic DNA was isolated from peripheral blood by using RelaxGene blood DNA isolation kit (Tiangen Biotech Co. LTD., Beijing, China).* CYBB*,* CYBA*,* NCF1*,* NCF2*, and* NCF4* gene mutations were analyzed using PCR amplification followed by direct sequencing. PCR reaction was performed in 30 cycles of 94°C for 15 seconds, 58°C for 30 seconds, and 72°C for 1 minute. Products were verified by 1% agarose gel electrophoresis and then sent for Sanger sequencing. Mutations were identified by alignment with standard sequence published in NCBI. Reverse chain was also sequenced for confirmation. High throughput exon sequencing was done in parts of patients and PCR followed by Sanger sequencing was conducted to verify the sequencing results.

### 2.5. Statistical Analysis

Statistical analysis was accomplished using SPSS 17.0 software. Significant differences were determined by an unpaired *t*-test. *P* value < 0.05 was considered as statistically significant.

## 3. Results

### 3.1. Population

A total of 48 CGD patients (44 male and 4 female) were enrolled in this study. These patients were distributed in 17 provinces and municipalities throughout Mainland China. The first CGD case diagnosed in our PID center was in 2005, while most of the patients (98%, 47/48) were diagnosed after 2007 and 75% (36/48) patients were diagnosed after 2010 (Table 1).

The 48 patients were from 48 unrelated nonconsanguineous families. Family history information was obtained from 47 of 48 patients, while P35 was an adopted child, whose family history information could not be collected. 13 of 47 patients (28%) had positive family histories, which means early death histories in other family members within three generations. Of the 13 patients, 8 (P10, P20, P23, P25, P27, P31, P43, and P46) had family histories of suspected PID in the first generation. All except P43 had elder brothers' death at an early age. P43 had an elder sister who died due to systemic infection at 3 years old. Four patients (P22, P27, P33, and P39) were reported to have family histories in the second generation, and all of them had maternal uncles' death at an early age. No patient had third-generation family history. The other 34 patients (71%) had no family histories (Table 1). Among the 13 patients with positive family histories, 11 patients carried* CYBB* gene mutations, P43 carried* NCF2* gene mutation, and P46 had unknown gene mutation.

### 3.2. Clinical Courses

**Figure 1:**
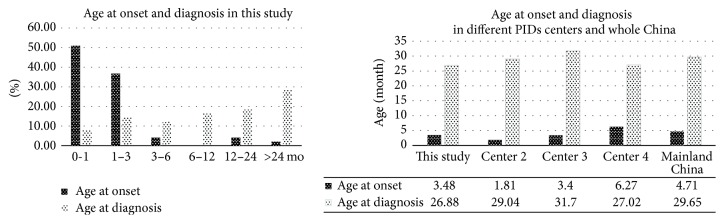
The age at onset and diagnosis of CGD in this study and other studies reported from Mainland China.

The mean onset age of 48 CGD patients was 0.29 ± 0.66 years old (median 0.83 years old, range 0–3.5 years old). Most of the patients (90%, 43/48) had an onset within the first 3 months of life and over half (52%, 25/48) of them had an onset within the 1st month of life. Of them, 13% (6/48) patients had an onset after birth. Only 1 patient (P40) had an onset of symptoms after 2 years old (3.5 years old) (Table 1 and Figure 1). The mean age at onset was 0.23 ± 0.45 years old in 36 XL-CGD patients and 0.16 ± 0.15 years old in 3 AR-CGD patients. There was no significant difference between them (*t* = 0.257, *P* = 0.799).

The mean age at diagnosis was 2.24 ± 2.98 years old (median 1 year old, range 0.07–15.00 years old). About one-half of patients were diagnosed after 1 year old (Table 1 and Figure 1). The mean diagnosis age was 2.38 ± 3.16 years old in 36 XL-CGD patients and 0.43 ± 0.28 years old in 3 AR-CGD patients. There was no significant difference between them (*t* = 1.059, *P* = 0.296).

The average lag time between onset of symptoms and diagnosis in CGD patients was 1.95 ± 2.75 years (median 0.75 years, range 0.03–13 years), which indicated there were obvious time lags between onset and diagnosis of CGD. Of these patients, 36 XL-CGD patients had an average lag time of 2.16 ± 2.91 years, while 3 AR-CGD patients had an average lag time of 0.27 ± 0.21 years. There was no significant difference between them (*t* = 1.109, *P* = 0.275).

To date, 11 patients (22%) had died with an average age of 2.91 ± 3.77 years old (median 1 year old, range 0.5–13 years old), 7 of whom (64%, 7/11) died before 1 year old. Among the 11 patients, 10 (91%) carried* CYBB* gene mutations; only P47 (9%) had unknown gene mutation. All of the 11 patients died due to infections, including severe pneumonia (3 patients, P1, P33, and P47), septicopyemia (3 patients, P23, P25, and P29), pulmonary tuberculosis (1 patient, P3), osteomyelitis (1 patient, P13), liver abscess (1 patient, P35), necrotic enteritis (1 patient, P36), and infections after haematopoietic stem cell transplant (HSCT) (1 patient, P30). The other 37 patients were alive with an average age of 6.59 ± 3.95 years old (median 6.5 years old, range 1–20 years old) (Table 1).

### 3.3. Infectious Clinical Manifestations

In our cohort study, the most prevalent infectious sites were the lungs, followed by gastrointestinal tract, lymph nodes, and skin (Table 2).

Pulmonary infections were observed in most of CGD patients (77%, 37/48), including pneumonia, pulmonary tuberculosis, and bronchitis. Pneumonia was the most common pulmonary infection, occurred in 73% (35/48) CGD patients. 10 of 35 patients were reported to have recurrent pneumonia at least 2 episodes, 3 of whom (P7, P15, and P17) had recurrent pneumonia with an average of 10, 4, and 2 episodes per year, respectively. Of the 37 cases, 6 were diagnosed with fungal pneumonia (P6, P15, P18, P20, P28, and P37). Pathogenic microorganism was isolated from 6 patients, including* Aspergillus *spp. from P6, mold from P28,* mycoplasma *spp. from P14,* Viridans streptococci *from P4*, Staphylococcus aureus *and* Enterobacter aerogenes* from P20, and* Acinetobacter baumannii* from P24. Pulmonary tuberculosis (TB) was reported in 7 patients, and all of them were considered to be caused by BCG infections. That is because they all had adverse reactions to BCG but did not exposure to* mycobacterium tuberculosis*. However,* mycobacterium tuberculosis *culture was not done in this study. Of the 7 cases, 2 (P3 and P34) had pulmonary TB alone, and 5 (P4, P6, P8, P21, and P28) were complicated by pneumonia. Besides, bronchiolitis was reported in P42, who was complicated by pneumonia. In addition to LRIs, upper respiratory infections (URIs) were reported in CGD patients previously. In our single center study, 6 patients (P7, P15, P21, P22, P36, and P37) were reported to have recurrent URIs (*⩾*5 episodes per person per year), 3 of whom had URIs more than 10 episodes per person per year, and all of the 6 patients presented with upper respiratory infection as initial symptom. Of the 6 patients with recurrent URIs, 5 (P7, P15, P36, and P37) were complicated by pneumonia, and P21 was complicated by pneumonia as well as TB. Besides, maxillary sinusitis was observed in P17, who was complicated by pneumonia (Table 1).

After the lungs, gastrointestinal tract was most vulnerable to being affected (54%, 26/48). The most frequent disease was perianorectal abscess (18 patients, 38%), 5 patients were treated with abscess excision. Of them, 4 patients recovered after the surgeries, only P35 had relapse. The remaining 13 patients took conservative treatments, most of whom (92%, 12/13) had protracted course.* Burkholderia Cepacia *was isolated from P23. Of the 18 patients, 8 were complicated by recurrent diarrhea, 1 by liver abscess (P31), and 1 by recurrent diarrhea as well as liver abscess (P46). A significant number of patients (15 patients, 31%) suffered from recurrent diarrhea, and most of them had an early onset after birth, with an average of 3.47 ± 2.27 months old (range 0.07–6 months). In the 15 patients, P46 had diarrhea due to intestinal flora imbalance after intensive use of antibiotics, P2 due to* Pseudomonas aeruginosa* infection. P10 and P19 were diagnosed with rotavirus enteritis, P6 with bacillary dysentery, P36 with necrotizing enteritis. The recurrent diarrhea gradually improved in all the 15 patients, and the latest case (P6) had his symptomatic relief at 3 years old. In the 15 patients, 8 were reported to have anemia caused by diarrhea, including mild anemia in 5 patients (P4, P10, P19, P26, and P29) and moderate anemia in 3 patients (P5, P15, and P38). Besides, liver abscesses were reported in 4 patients (8%, P13, P21, P31, and P46) in this study.* Staphylococcus aureus* was isolated from P13, who had recovery after drainage of liver abscess and anti-infective therapy. P21 was diagnosed with tuberculous liver abscess due to BCG infection (Table 1).

Lymphnoditis (50%, 24/48) was also commonly reported in this study (Table 1), and most of the cases (79%, 19/24) were caused by BCG infections. Of the 19 cases, enlarged axillary lymph nodes were observed in 15 cases (78.95%, 15/19), followed by cervical ones in 3 cases (16%, 3/19) and an inguinal one (5%, 1/19). P29 suffered from lymphnoditis in his left axilla due to BCG infection; then lymph node abscess was observed in his left groin. Of the remaining 5 cases with lymphnoditis not due to BCG infections, inguinal lymphnoditis was observed in 3 cases (P17, P24, and P30) and submandibular lymph node abscesses in 2 cases (P36 and P40).* Staphylococcus aureus* and* burkholderia cepacia* were isolated from P40 and P30 after pus cultures. All of the 5 cases had recovery after surgical drainage.

Skin was the fourth frequently affected site of this disease (22 patients, 46%). Skin abscesses were observed in 11 patients (23%, 11/48) with late onsets, 7 of whom (64%, 7/11) had their onsets after 5 years old. P39 was diagnosed with cutaneous TB abscess. Most of the skin abscess were located at arms, iliaca, face and ear. Pustular eruption were observed in 10 patients (21%, 10/48), 8 of whom (80%, 8/10) had an onset within the 1st week of life. P14 was diagnosed with orbital cellulitis on the right side combined with dacryocystitis, and the cut of the lacrimal sac had a granulomatous change. Besides the infectious symptoms mentioned above, some noninfectious symptoms were also observed in this study. Eczema was reported in 8 patients in this study. Of them, P39 suffered from eczema complicated by skin abscesses, and P27 suffered from eczema complicated by pustular eruption. Four patients (P5, P14, P31, and P41) were reported to have skin granulomas. P5 suffered from skin granulomas complicated by skin abscess (Table 1).

Septicopyemia was also reported in CGD patients previously. In the present study, 11 patients (23%) suffered from septicopyemia, and treatments with antibiotics were effective for most of these cases (73%, 8/11), while the remaining 3 patients died of septicopyemia (P23, P25, and P29). Other severe infections, such as central nervous system (CNS) infections, organ abscesses were less reported in this study. CNS infections were reported in P18 and P27, with purulent meningitis and encephalitis, respectively. Splenic abscess, left kidney abscess, and testicular abscess was observed in P41, P2, and P22. P13 was diagnosed with osteomyelitis due to* Acinetobacter baumannii* infection.

Besides, thrush and otitis media were reported in 23% (11/48) and 8% (4/48) patients, respectively. Six patients (P6, P10, P24, P28, P33, and P36) were still having thrush after 1 year old. Other infections less occurred in our study. Virus infections were observed in 4 cases, including Epstein-Barr virus (EBV) infection in P4 and P17, respiratory syncytial virus (RSV) infection in P11, and cytomegalovirus (CMV) infection in P13. Besides, urinary tract infection was reported in P14.

Infection-related symptoms also occurred in this study. Hepatosplenomegaly was the most common infection-related symptom, accounting for 40% (19/48) cases. Most of them (95%, 18/19) were complicated by liver damage. Hepatomegaly was reported in 17% patients (8/48), and all of them were complicated by liver function damage (Table 1).

### 3.4. Other Clinical Manifestations

Anemia was observed in 11 patients (23%) in this study, including mid anemia in 7 patients and moderate anemia in 4 patients. Of the 11 patients, 8 had anemia due to diarrhea. Congenital heart disease was reported in a significant number of patients (13%, 6/48), including atrial septal defect in P20, atrial septal defect complicated by patent ductus arteriosus in P3, patent foramen ovale in P9 and P24, ventricular septal defect in P10, double arches, and left arch atresia complicated by tracheostenosis in P37.

Allergic diseases were also reported in the present study. Eczema occurred in 8 patients. P13 suffered from eczema as well as allergic rhinitis. P4 suffered from eczema as well as hypereosinophilia (0.88 × 10^9^/L). Besides, urticaria was reported in P17.

Autoimmune disease was reported in 3 patients, including Kawasaki disease in P10 and P38 and Behçet's disease in P28. P28 had an onset at 6 years old, presenting with recurrent oral ulcer and fever. He suffered from recurrent skin and joint pains and then ulceration at 8 years old, which responded well to hormone therapy.

Other noninfections were rarely observed in this study. Hydrocele was reported in P14, left renal cyst in P31, vitamin K deficiency in P11, severe malnutrition and neonatal hyperbilirubinemia in P18, and acanthosis nigricans in P17.

### 3.5. BCG Complications

Of the 48 GD patients, 45 patients (94%) received BCG vaccination after birth according to a national vaccination program, 1 patient (P18, 2%) did not received BCG vaccination because of neonatal hyperbilirubinemia, and the remaining 2 patients (P16 and P37, 4%) had unknown vaccination histories. In the 45 BCG-vaccinated CGD patients, more than half (53%, 24/45) suffered from BCG infections. The most frequent pattern was distant infection (20 cases), followed by regional infection (17 cases) and disseminated infection (2 cases). Of them, 12 patients were reported to have both regional and distant infections. Disseminated infection was observed in P8 and P21 (4%) (Table 1). P8 had lymph node enlargement in his groin and right axilla, complicated by TB in bone and lung. P21 had enlarged lymph nodes in his left armpit and liver tuberculosis 3 months later after BCG vaccination, thus was given regular anti-TB treatment. Six months later, he was infected with measles virus, followed with dissemination of TB infection to other sites (lung, lymph nodes, and mediastinum). The most common sites of occurrence for distant infections were lymph nodes (41%, 19/46), followed by lungs (15%, 7/46).

### 3.6. Immunological Characteristics

Peripheral leukocyte subsets and immunoglobulin levels were analyzed in most of the patients in this study (Table 3). White blood cell (WBC) counts were high in most of the patients (88%, 42/48), while the remaining 6 patients (12%) had normal counts. Likewise, neutrophils were elevated in most of the patients (92%, 44/48). Respiratory burst activation in neutrophils was measured in most of the patients in this study (34/48, 71%), and all of them had significantly lower neutrophil oxidative function compared to healthy controls. Besides, although the percentages of lymphocytes were present in normal range in most of the CGD patients (73%, 35/48), the counts of lymphocytes were high in over half of patients (52%, 25/48). T cells, B cells, and NK cells were reported to be normal in most of the patients tested, with a percentage of 98%, 93%, and 93%, respectively. In addition, high serum IgG levels were found in most of the patients (88%, 42/48). IgG levels were normal in the other 6 patients.

### 3.7. Genetic Analysis

Genetic analyses were performed in all of the 48 CGD patients in the present study. 39 mutations were found in 48 patients, including 36 mutations in* CYBB* gene, 1 mutation in* CYBA* gene, 1 mutation in* NCF1* gene, and 1 mutation in* NCF2* gene.

For* CYBB* gene, 17 missense, 5 nonsense, 7 deletion, 1 insertion, and 6 splicing error mutations were identified. Among them, 11 novel mutations were reported, including 5 missense mutations (c.376T>C in P15, c.1414G>A in P17 c.1328G>A in P20, c.911C>G in P28 and c.184T>A in P30), 3 deletions (c.616delT in P19, c.218delG in P24 and c.185_186delTC in P33), 1 insertion (c.1206_1208insGGT in P1), and 2 splicing errors (c.1315A>G in P22 and c. [898-2_902 AGGTGGTdel] in P32). A female carrier of X-linked CGD (P17) was found to have a heterozygous mutation in CYBB gene (c.1414G>A). The frequency of the A variant at c.1414 in CYBB gene is 2.1‰ in 1000 genomes and 6.3‰ in Chinese Han population. The female patient with this heterozygous mutation in our study had defective NADPH oxidase activity and clinical symptoms consistent with CGD. Further exon sequencing was done in this patient, but no other CGD-related gene mutations was found. These above indicated that the G to A transition at position 1414 in* CYBB* gene and an extremely skewed X-inactivation event resulted in CGD phenotype in this female patient.

One patient was found to have* CYBA* gene mutation. A homozygous missense mutation (c.7C>T), which has been reported in other CGD patients in China previously, was also identified in P37 in our study. P41 was found to have a compound heterozygous mutation in* NCF1* gene (c. [541delG; 923T>C]). In the* NCF2* gene, a homozygous missense mutation (c. 550C>T) was found in P43 (Table 5).

## 4. Treatment and Outcome

In our PIDs center, all the patients were recommended to have preventive treatment with cotrimoxazole and itraconazole to prevent bacterial and fungal infections. IFN-*γ* therapies were used when the CGD patients had severe infections. Only 13% patients (6/48) had ever received IFN-*γ* treatment. Administrations of IFN-*γ* were reported to be effective in reducing infections in 67% patients (4/6, P7, P19, P28, and P33). By the end of this study, 4 CGD patients had been treated with haematopoietic stem cell transplantation (HSCT). One patient (P30) died because of serious infections after transplantation; the other 3 patients (P10, P19, and P34) were still alive at the time of inclusion.

## 5. Discussion

CGD is a rare primary immunodeficiency, which is characterized by the defect in phagocyte respiratory burst oxidase and thus recurrent infections [6, 20, 34, 35]. The incidence of CGD varied between countries. However, there was no national PID registration system in China and therefore no reliable prevalence data on Chinese CGD patients. Until now, a total of 205 CGD patients (48 patients in this study and 157 patients published previously) were reported from Mainland China. Most of the cases (86%, 176/205) were from four major PID centers in Mainland China, including 48 patients from our center, 38 from center 2 [15], 48 from center 3 [16, 20] and 42 from center 4 [21–30]. If we calculated according to the China population of 1,360,000,000 persons and the incidence of CGD in Japan (1/300,000) and the United States (1/200,000), an estimate of approximately 4533 and 6800 persons with diagnosed CGD in China. However, there were only 205 CGD patients were reported from Mainland China, which were far less than other countries. This indicated that CGD patients might be underdiagnosed in China. The imbalance of medical resources in China and the lack of recognition of PID in primary hospitals may contribute to the underdiagnosis of CGD. Furthermore, for the proportion that CGD accounting for PIDs, it was 14% in Japan [36], 9% in the United States [37] and 6% in Taiwan [38]. In our single center study, there were 48 patients diagnosed with CGD, accounting for 8% in 575 PIDs patients, similar with the United States, while the proportion was 4% (5/138) in another single center study in Mainland China [17]. Unfortunately, it is hard for us to estimate the national prevalence data on CGD because of lacking reliable data, and further work is still needed to clarify it.

The first CGD case reported in Mainland China was in 1981 without gene analysis [39], while most of patients were diagnosed after 2004. This indicated that people gradually paid more attention to PID in China.

According to the previous CGD cohort studies from other countries, XL-CGD caused by* CYBB* gene mutations accounted for about 70% CGD cases. For instance, XL-CGD accounted for 74% CGD in Japan, 74% in the United States [10] and 68% in European series [11, 12]. However, the proportion did not apply to the regions where consanguineous marriage was permitted, such as Turkey and Iran [32, 40]. The high rate of consanguineous marriage led to a higher rate of AR-CGD. In our single center study, XL-CGD subgroup accounted for 75% cases, similar with other single center studies in China (79% in Center 2 and 65% in Center 3) (Table 6). Besides, the ratio of male to female was 11 : 1 in this study, similar with all the 205 CGD patients in Mainland China (12 : 1; 189 male and 16 female). However, the ratio of male to female patients was higher in the United States (6.08 : 1; 316 male and 52 female) [10] and Japan (6.6 : 1; 199 male and 30 female) [12]. This indicated that female CGD patients were underdiagnosed in Mainland China. The reasons for the underdiagnosis for female may be as follows. (1) There was a bias for Chinese physicians towards boys as increased risk of being CGD. (2) Most of female patients were AR-CGD patients, and Chinese physician had insufficient recognition of AR-CGD, which occurred not only in the primary hospital, but also in the tertiary hospital. In addition, in our single center study, 28% patients had positive family histories. For all the 205 CGD patients reported in Mainland China, 33 patients were excluded because of lacking reliable information from the published paper; of the remaining 172 patients, 47 patients (27%) had positive family histories, similar with our single center study.

In our single center study, the mean age at onset/diagnosis was 3.48/26.88 months old. There was a long diagnosis lag between the onset and diagnosis of this disease (Figure 1). For all the patients from Mainland China, 198 had available information about onset age, with a mean age of 4.73 months old; 200 patients had available information about diagnosis age, with a mean age of 29.69 months old, similar with our single center study. However, both of the mean onset and diagnosis age in CGD patients from other countries were later than Chinese patients [10, 11, 41]. A possible explanation was that BCG vaccinations were given routinely to all Chinese newborns at birth as part of the National Vaccination Programs. CGD patients were susceptible to BCG infections thus had early onsets. Besides, as a developing country, the serious environment pollutions in China might also contribute to the early onsets in CGD patients. Furthermore, almost all CGD patients reported in Mainland China were children, and there was an obvious bias for Chinese physicians towards children, especially boys as increased risk of being CGD. This bias not only made the female and adult CGD patients underdiagnosed but also lowered the mean age at onset and diagnosis. Finally, milder clinical course in NCF1 mutant patients and the insufficient recognition of NCF1 mutant patients in Chinese physicians made the statistical data on onset and diagnosis age earlier.

It has been reported that mutations in* NCF1* gene led to milder course and later age at onset of CGD; however, the other autosomal forms of this disease were as serious as the X-linked form [32]. In fact, because NCF1 mutant patients accounted for majority of AR-CGD patients, and many studies have showed that XL-CGD patients had earlier age at onset and diagnosis [11, 41]. However, onset/diagnosis age in XL-CGD and AR-CGD had no significant difference in our single center study. The reasons for the difference were probably as follows: (1) statistical limitation because of the small samples and (2) the underdiagnosis of* NCF1* mutant patients. Only patients with severe clinical manifestations were diagnosed at early ages, while many patients with milder clinical manifestations were not, which made the average age at onset and diagnosis in NCF1 mutant patients earlier. Besides, 22% of patients died up to the time of inclusion in this study, little higher than Italy and Europe (13% and 20%, resp.). However, the median age at death was 1 year old in this study, significantly lower than Italy and Europe (8.8 and 10.4 years old, resp.) [11, 41]. This reminds us that more efforts are still needed to improve the outcomes of CGD patients in Mainland China.

CGD patients were reported to suffer from recurrent and even life-threatening infections [5]. Infections which occurred in CGD patients from 4 major PID centers in Mainland China were summarized in Table 2. Similar with previous reports, the infectious sites of this disease were mostly lung, gastrointestinal tract, skin/subcutis, and lymph nodes in Chinese patients. However, for the incidence of different infections, there were still some differences between different centers. For example, the incidence of pneumonia was 73% in our study, while it is 84% in center 2, 92% in center 3, and 81% in center 4, respectively.

Lung was the most common infectious site reported in all 4 center studies. Of the lung infections, pneumonia was the most prominent infection observed in our single study. Pneumonia was also the most common infection reported in other 3 PID center studies, with an incidence of 84% in center 2, 92% in center 3, and 81% in center 4, respectively. “Sampling bias” was the likely explanation for the different incidence between different studies. For all 176 CGD patients from 4 PID centers, the incidence of pneumonia was about 82%, similar with United States and Japan (with an incidence of 79% and 88%, resp.) [10, 13]. However, pneumonia was less commonly reported in some European countries, with an incidence of 67% in Germany [42], 47% in Italy [11, 41], and ⩽66% in European series [11] (Table 2). Fungal pneumonia, which was reported in CGD patients from other countries [10, 11], was also reported in this study. However, the fungal pathogens detection rate was low in this study.* Aspergillu*s spp. were only isolated from 1 case. In fact,* Aspergillu*s spp. were reported to be the most common microorganism isolated from CGD patients with pneumonia, with an incidence of 41% in the United States and 18% in Europe series [10, 11]. The low positive pathogenic microorganism detection rate was partially due to delaying or lacking of microorganism detection and low-threshold use of antibiotics in primary hospital in Mainland China. Besides, 14% patients were reported to suffer from pulmonary tuberculosis in this study, similar with center 3 (*⩾*21%) and center 4 (14%) [20]. However, the incidence of pulmonary tuberculosis was very high in center 3 (55%) [15] (Table 2). In fact, our center and center 3 were both located at Shanghai; center 4 was located at Beijing. Both of the cities had special hospitals to receive patients with pulmonary tuberculosis, and patients with pulmonary tuberculosis had to be transferred to these hospitals, which was the probable explanation for the lower case finding in these 2 cites. Lung abscess, another type of lung infection, which was reported in CGD patients previously, was not found in this study and was rarely found in other studies in China. It was also less reported in some western countries (3% in Italy and 6% in European series) [11, 41]. However, a considerable number of patients (16%) suffered from lung abscess in the United States [10] (Table 2).

After the lung infections, GI disease was also very common in this study. It was also commonly reported in other studies (40% in center 4 and 48% in Europe). For perianal abscess, 48 patients from center 2 had no available data from the published paper. Of the remaining 138 patients from the other 3 PID center studies, the incidence of perianal abscess was 28% (39/138), which was higher than United Kingdom and Ireland (15%), Italy (8%), and European series (21%) [11, 41, 43]. Likewise, Chinese CGD patients had higher risk of getting diarrhea. Notably, although liver abscess was reported to affect quite a number of CGD patients (46% in Japan [10], 27% in the United States, and 32% in European series), it was less reported in Chinese CGD patients (Table 2). The reasons for the low incidence were still to be determined. Another frequent clinical manifestation of CGD, lymphadenitis, was also commonly observed in our study (51%), most of which (80%) were cause by BCG infections. The incidence of lymphadenitis was similar in different studies (Table 2).

Skin/subcutis infections were commonly reported in our study, similar with other Chinese single center studies (46% in center 3 and 40% in center 4), the United States (42%), and Europe (53%). Skin abscess was the most predominant skin infection in CGD patient in our single center study. However, the incidence of skin abscess varied between different Chinese single center studies, accounting for 42% in center 2, 23% in center 3, and 5% in center 4, respectively. The average incidence in 4 PID centers was 23% (40/176), which was similar with European series (17%) and Italy (20%) [11, 41] (Table 2).

Septicopyemia was also commonly observed in CGD patients previously, and it was an important cause of death. In our single center study, 3 of 9 patients died due to septicopyemia, similar with the United Kingdom and Ireland (18%) and European series (20%) [11, 43]. Another type of severe infections, osteomyelitis, was reported to occurred in a significant number of CGD patients previously (with the incidence of 25%, 13% and 16% in the United Kingdom and Ireland, Europe and Italy, resp.) [11, 41, 43], was less reported in Chinese CGD patients. Besides, brain abscess, which occurred in 3% CGD patients in the United States and 7% in the European series, was also rarely observed in Chinese CGD patients (Table 2).

China has a serious epidemic of TB. According to a national survey of tuberculosis prevalence in 2000, the incidence of TB in China was 367 in 100,000 person. In fact, routine BCG vaccination in many countries with TB burden is compulsory. In China, all newborns must be given BCG vaccination at birth. A markedly high incidence of BCG disease in our cohort study was observed. BCG disease was also commonly reported in other CGD cohort studies, with an incidence of 53% (17/32) in center 2, 47% (8/17) in Hong Kong, and 65% (22/34) in France [11, 15]. This high incidence of BCG disease was due to the defective respiratory burst of neutrophils in CGD patients. In addition, CGD patients were reported to be susceptible to TB. In our study, 41% patients suffered from TB, similar with another 2 Chinese cohort studies (55% in center 2 and 41% in Hong Kong) [15].

Summary of inheritance pattern of CGD patients in different large cohorts was shown in Table 6. Although mutation detection rate varied in different countries because of different healthcare level and genetic sequencing technology, it is clear that XL-CGD patients caused by gp91^phox^ deficiency made up majority of CGD patients, ranging from 65% to 70%. Up to date, 718 different kinds of mutations in* CYBB* gene were reported in HGMD data base (http://www.hgmd.cf.ac.uk/ac/gene.php?gene=CYBB). In Mainland China, a total of 89 different* CYBB* gene mutations were reported in 138 patients, including 36 patients in this study and 102 patients published previously in English and Chinese versions. Of them, mutations in 15 patients were not included because of lacking detailed mutation information from the published papers; the other 123 mutations in* CYBB* gene were summarized in Table 4. 36 missense, 21 nonsense, 36 splicing error, 29 deletion and 1 insertion mutations were included. These mutations were showed to be distributed over almost all exons, and the most distributed exon was exon 9, followed by exon 6. No mutation was found in exon 4. Missense, splicing error, deletion, and nonsense mutations were common mutation types found in Mainland China, accounting for 30%, 30%, 24%, and 17%, respectively, which was similar with other reports. However, insertion mutations, which were commonly reported in other CGD cohort studies, were less found in Mainland China [12]. In addition, gross deletions were found in 8 patients, including 5 whole* CYBB* gene deletions. Notably, a female CGD patient with* CYBB* gene mutation was reported in our study, which is the first report of female CGD patient with gp91^phox^ deficiency in Mainland China (Table 4).

In regard to the AR-CGD, which is mostly caused by* CYBA*,* NCF1*, and* NCF2* gene mutations, they accounted for about 30% CGD cases according to the previous study from western countries [6]. However, AR-CGD was less reported in Mainland China. Up to now, only 6 patients with* CYBA* gene mutations, 5 patients with* NCF1* gene mutations, and 3 patients with* NCF2* gene mutations were reported from Mainland China. These mutations were summarized in Table 5. The low case finding rate was possibly because of the insufficient recognition of AR-CGD in Chinese physicians, which made the patients lost the chance to detect gene mutations. Another possible reason was that the consanguineous marriage was strictly forbidden in China, which lowered the prevalence of AR-CGD. With the development of the next sequencing technology in China, a rapid genetic diagnosis becomes realizable, thus greatly promotes genetic diagnosis of PID. In addition, it has been reported that* NCF1 *gene mutations were the most common causes leading to AR-CGD, which occurred in over 20% CGD in western countries [6]. However, the incidence seems to be lower in Asia countries, with an incidence of 2% and 7% in our study and Japan, respectively [12]. The reason for the different incidences is still to be determined.

CGD was originally termed “fatal granulomatous disease of childhood” [44], which indicated that it had high lethality. With the development of medical level, the prognosis of CGD have improved over time. At present, it is widely accepted that CGD patients should take lifelong antibacterial and antifungal prophylaxis and interferon gamma (IFN-*γ*) treatment [45]. In fact, the treatment using INF-r is still controversial. Some studies suggested that IFN-*γ* could effectively reduce the number and severity of infections, while other studies showed IFN-*γ* treatment did not reduce the infections [41, 46]. In our single center study, only 6 patients have been treated with IFN-*γ*, and it was effective in 67% of them. However, this was unrepresentative because of the small sample and lacking long-term follow-up study. In fact, recombinant human IFN-*γ* is a very expensive therapeutic agent, which made it difficult to be applied widely. Therefore, the effect of IFN-*γ* treatment on Chinese CGD patients still need to be determined.

Up to now, HSCT is the only proven curative treatment for CGD [45]. However, because of the high survival rate of CGD patients, high cost of HSCT, relapse, and high risk of infections after HSCT, most patients in our study did not choose HSCT treatment. Similarly, only 3 CGD patients (8%) in another Chinese cohort study received HSCT [15]. Notably, as a single gene disorder, gene therapy is a potential treatment for CGD, which provides new hope for cure of CGD.

## 6. Conclusions

We here report 48 CGD patients in our single center study, which is the largest cohort study from Mainland China. 39 gene mutations were identified in 48 patients, including 36 mutations in* CYBB* gene, 1 mutation in* CYBA* gene, 1 mutation in* NCF1* gene, and 1 mutation in* NCF2* gene. CGD patients are susceptible to BCG infections and they should avoid BCG vaccination. The compulsory BCG vaccination for all infants after birth contributes to the early onsets in CGD patients in Mainland China.

## Figures and Tables

**Table 1:** Clinical characteristics of 48 CGD patients in this study.

Pts/ sex	Year of diagnosis	Family history	Onset age (mo)	Diagnosis age (mo)	Status-age (death cause)	Type of infections										BCG disease			SI
						Lung		Skin		Digestive system			Lymphadenitis	Septicopyemia	Other symptoms	Regional	Distant	Disseminated	
						Pneumonia	TB	Pustular eruption	Skin abscess	Perianal abscess	Diarrhea	Liver abscess							
P1/M	2015	−	0	36	Dead-3 y (severe pneumonia)	+	−	+	−	+	−	−	+	−	UTI, HSM	+	+	−	ND
P2/M	2010	−	2	24	Alive-9 y	−	−	−	−	−	−	−	−	−	Left renal abscess, left hydronephrosis, hypospadias	−	−	−	ND
P3/M	2013	−	1	7	Dead-1 y (TB)	−	+	−	−	−	−	−	+	−	ASD, PDA	+	+	−	18
P4/M	2014	−	3	17	Alive-3 y	+	+	−	−	−	+	−	+	+	Mild anemia, eczema, EBV infection, eosinophilia, otitis media	−	+	−	1.27
P5/M	2013	−	3	12	Alive-5 y	+	−	−	+	+	+	−	−	+	Moderate anemia, skin granulomas, HSM	−	−	−	ND
P6/M	2012	−	0	72	Alive-11 y	+	+	−	−	+	+	−	+	−	HSM, thrush	+	+	−	2.95
P7/M	2014	−	2	48	Alive-7 y	+	−	−	+	+	−	−	−	−	Recurrent URI, HSM	+	−	−	4.57
P8/M	2005	−	0	72	Alive-17 y	+	+	+	−	−	−	−	+	−	HSM	+	+	+	ND
P9/M	2013	−	2	7	Alive-3 y	+	−	−	−	−	−	−	−	−	PFO	−	−	−	ND
P10/M	2010	+	2	3.5	Alive-7 y	−	−	−	−	−	+	−	−	+	Mild anemia, eczema, Kawasaki disease, Thrush, ventricular septal defect	−	−	−	1.41
P11/M	2014	−	0	24	Alive-4 y	+	−	−	+	−	−	−	+	−	Vitamin K deficiency	+	+	−	ND
P12/M	2015	−	1	36	Alive-4 y	−	−	+	−	−	−	−	+	−	HSM	−	+	−	ND
P13/M	2007	−	0.93	36	Dead-4 y (osteomyelitis)	+	−	−	−	−	−	+	−	−	Osteomyelitis, urticaria, eczema, thrush	+	−	−	0.76
P14/M	2011	−	2	36	Alive-9 y	+	−	−	−	−	−	−	+	−	Orbital cellulitis, dacryocystitis, skin granulomas, otitis media, HSM	+	+	−	0.96
P15/M	2012	−	6	24	Alive-7 y	+	−	−	−	−	+	−	−	−	Moderate anemia, recurrent URI, thrush	−	−	−	3
P16/M	2015	−	1	3	Alive-1 y	−	−	+	−	+	+	−	−	−	HSM	NA	NA	NA	ND
P17/F	2015	−	3	48	Alive-5 y	+	−	−	−	−	−	−	+	−	AR, MS, AH, AN, moderate anemia, EBV infection	−	−	−	24.64
P18/M	2015	−	0.67	1	Alive-1 y	+	−	−	−	−	−	−	−	+	Severe malnutrition, myocardial injury, NH, purulent meningitis, HM	NA	NA	NA	ND
P19/M	2014	−	3	6	Alive-3 y	+	−	−	−	−	+	−	+	−	Mild anemia, HSM	−	+	−	0
P20/M	2012	+	0	2	Alive-4 y	+	−	+	−	+	−	−	−	+	ASD, HSM	−	−	−	ND
P21/M	2014	−	3	18	Alive-4 y	+	+	−	−	−	−	+	+	−	HSM, recurrent URI	−	+	+	0
P22/M	2011	+	1	10	Alive-6 y	−	−	−	+	+	+	−	−	−	Testicular abscess, otitis media, thrush recurrent URI, HM	−	−	−	0.86
P23/M	2011	+	1	2	Dead-1 y (septicopyemia)	+	−	−	−	+	+	−	−	+	HM	−	−	−	0.94
P24/M	2012	−	2	18	Alive-6 y	+	−	−	−	+	−	−	+	−	Hydrocele, heart failure, PFO, thrush	+	−	−	ND
P25/M	2009	+	2	4	Dead-1 y (septicopyemia)	−	−	−	−	+	−	−	−	−	HSM	−	−	−	0.77
P26/M	2008	+	0.33	1	Alive-9 y	+	−	+	−	−	+	−	+	−	HSM, mild anemia	+	+	−	0.74
P27/M	2009	+	0.1	2	Alive-8 y	−	−	+	−	+	−	−	−	−	Eczema, brain edema, encephalitis, HM	−	−	−	1.59
P28/M	2011	−	24	180	Alive-20 y	+	+	−	+	+	−	−	+	−	Behçet's disease, thrush	+	+	−	ND
P29/M	2013	−	1	6	Dead-0.5 y (septicopyemia)	+	−	−	−	+	+	−	+	+	HSM, mild anemia	−	+	−	2.73
P30/M	2008	+	0.67	3	Dead-0.5 y (infections after HSCT)	−	−	+	−	−	−	−	+	−	HSM	−	−	−	1.99
P31/M	2009	−	2	24	Alive-9 y	+	−	−	−	+	−	+	+	+	Renal cyst, mild anemia, skin granulomas, HSM	−	+	−	0
P32/M	2013	−	0.5	1	Alive-3 y	+	−	−	+	−	−	−	−	−		−	−	−	4.25
P33/M	2010	+	3	132	Dead-13 y (severe pneumonia)	+	−	−	−	−	−	−	+	−	Eczema, thrush	+	+	−	0.72
P34/M	2015	+	0	48	Alive-6 y	−	+	−	−	−	−	−	+	−	Hypokalemia	+	+	−	3
P35/M	2012	NA	0.07	6.63	Dead-1 y (liver abscess)	+	−	−	−	+	+	−	+	−	HM	+	+	−	2.75
P36/M	2007	−	24	60	Dead-6 y (NEC)	+	−	−	+	−	+	−	+	+	HM, recurrent URI, thrush	−	−	−	16.70^$^
P37/M	2014	−	4	8	Alive-2 y	+	−	−	−	−	−	−	−	−	HSM, CHD, recurrent URI	NA	NA	NA	ND
P38/M	2013	−	3	12	Alive-4 y	+	−	−	+	+	+	−	−	+	HSM, moderate anemia, Kawasaki disease, otitis media	−	−	−	24.67
P39/F	2010	+	0	24	Alive-8 y	−	−	−	+	−	−	−	−	−	HM, eczema, liver function damage	+	−	−	3.62
P40/M	2011	−	42	48	Alive-9 y	−	−	−	−	−	−	−	+	−	Eczema	−	−	−	1.83
P41/F	2014	−	0.67	6	Alive-3 y	+	−	−	+	−	−	−	−	−	Skin granulomas, splenic abscess	−	−	−	6.15
P42/M	2014	+	3	60	Alive-7 y	+	−	−	−	+	−	−	+	−	Mild anemia, tonsillar enlargement, BO, thrush	+	+	−	ND
P43/M	2005	−	1	1.5	Alive-11 y	+	−	−	+	−	−	−	−	−		−	−	−	4.27
P44/M	2011	−	2	24	Alive-7 y	+	−	−	−	−	−	−	−	−		−	−	−	5.43
P45/F	2011		0.4	0.83	Alive-5 y	−	−	+	−	−	−	−	−	−	Omphalitis	−	−	−	2.62
P46/M	2007	+	0.5	12	Alive-10 y	+	−	+	−	+	+	+	−	−	HM	+	−	−	1.99
P47/M	2007	−	3	5	Dead-1 y (severe pneumonia)	+	−	−	−	−	−	−	+	−	HSM, eczema, umbilical hernia	−	+	−	40.15
P48/M	2009	−	0.67	2	Alive-7 y	+	−	−	−	−	−	−	−	−	Thrush	−	−	−	0.94

Pts: patients; mo: month; TB: tuberculosis; BCG: Bacille Calmette-Guerin; UTI: urinary tract infection; ASD: atrial septal defect; PDA: patent ductus arteriosus; EBV: Epstein-Barr virus; PFO: patent foramen ovale; URI: upper respiratory infection; AR: allergic rhinitis; MS: maxillary sinusitis; AH: adenoid hypertrophy; AN: acanthosis nigricans; NH: neonatal hyperbilirubinemia; CHD: congenital heart disease; BO: bronchiolitis obliterans; HGM: hepatosplenomegaly; HM: hepatomegaly; NEC: necrotic enterocolitis; ND: not determined; NA: not available; SI: stimulation index.

^$^Besides the DHR assay, P37 also did 2 times of nitroblue tetrazolium (NBT) tests, and both of the results were 0%.

**Table 2:** Summary of infections in CGD patients from 4 major PID centers in Mainland China and 2 large cohort studies from other countries.

Infections	This study	Center 2	Center 3	Center 4	USA [10]	Europe [11]
	*n* = 48 (%)	*n* = 38 (%)	*n* = 48 (%)	*n* = 42 (%)	*n* = 368 (%)	*n* = 429 (%)
Lung	37 (77%)	—	44 (92%)	41 (98%)	—	284 (66%)
Pneumonia	35 (73%)	32 (84%)	44 (92%)	34 (81%)	290 (79%)	—
Lung abscess	0	—	4 (8%)	1 (2%)	60 (16%)	24 (6%)
Tuberculosis	7 (15%)	21 (55%)	*⩾*10 (21%)	6 (14%)	—	—
GI tract	26 (54%)	—	—	17 (40%)	—	208 (48%)
Diarrhea/enteritis	15 (31%)	14 (37%)	31 (65%)	7 (17%)	—	55 (13%)
Perianal abscess	18 (38%)	—	13 (28%)	9 (21%)	57 (15%)	88 (21%)
Liver abscess	4 (8%)	—	≥3 (6%)	1 (2 %)	98 (27%)	138 (32%)
Cutaneous/subcutaneous infections	22 (46%)	—	22 (46%)	17 (40%)	156 (42%)	229 (53%)
Pustular eruption	10 (21%)	—	9 (19%)	9 (21%)	—	—
Skin abscess	11 (23%)	16 (42%)	11 (23%)	2 (5%)	—	74 (17%)
Lymphadenitis	24 (50%)	16 (42%)	31 (65%)	26 (62%)	194 (53%)	213 (50%)
Septicopyemia	11 (23%)	—	—	16 (38%)	65 (18%)	85 (20%)
UTI	1 (2%)	—	3 (6%)	1 (2%)		52 (12%)
Bone	2 (4%)	—	2 (4%)	0		56 (13%)
Osteomyelitis	1 (2%)	—	1 (2%)	0	90 (25%)	56 (13%)
Bone tuberculosis	1 (2%)	—	1 (2%)	0	—	—
CNS	2 (4%)	—	—	6 (14%)	—	—
Meningitis	1 (2%)	—	—	5 (12%)	15 (4%)	—
Brain abscess	0	—	—	1 (2%)	12 (3%)	31 (7%)
Autoimmunity- rheumatology	3 (6%)	—	—	—	18 (5%)	26 (6%)

GI: gastrointestinal; UTI: urinary tract infection; CNS: central nervous system.

38 patients from center 2 were reviewed from 1 paper published in English version [15].

48 patients from center 3 were reviewed from 1 paper published in English version and 1 in Chinese version [16, 20].

42 patients from center 4 were reviewed from 10 papers published in Chinese version [21–30].

**Table 3:** Immunologic investigations in CGD patients in the present study.

	Patients tested (*n*)	Normal (*n*/%)	Low (*n*/%)	High (*n*/%)
WBC (10^9^ white blood cells/L)	48	6/13	0/0	42/88
Neutrophils (%)	48	2/4	2/4	44/92
Neutrophils (10^9^ neutrophils/L)	48	4/8	0/0	44/92
Lymphocytes (%)	48	35/73	10/21	3/6
Lymphocytes (10^9^ lymphocytes /L)	48	18/38	5/10	25/52
Eosinophils (%)	48	26/54	20/42	2/4
Eosinophils (10^9^ eosinophils /L)	48	29/60	19/40	0/0
CD3 T lymphocytes (%)	46	45/98	0/0	1/2
CD4 T lymphocytes (%)	46	38/83	7/15	1/2
CD8 T lymphocytes (%)	46	44/97	1/2	1/2
B lymphocytes (%)	46	43/93	2/4	1/2
NK lymphocytes (%)	46	43/93	0/0	3/7
IgG levels (g/L)	48	0/0	6/13	42/88
IgA levels (g/L)	48	35/73	11/23	2/4
IgM levels (g/L)	48	44/92	1/2	3/6
IgE levels (g/L)	42	32/76	0/0	10/24

**Table 4:** Summary of *CYBB* gene mutations reported in Mainland China.

Pts number	Mutation type	Exon/intron	Nt. change	AA. change	Reference
P1	Insertion	Exon 10	c.1211_1213dupGGT#	p.V404dup	This study
P2	Missense	Exon 12	c.1498G>A	p.D500N	This study
P3	Missense	Exon 5	c.466G>A	p.A156T	This study
P4	Missense	Exon 9	c.1085C>T	p.T362I	This study
P5	Missense	Exon 3	c.176G>T	p.C59F	This study
P6	Missense	Exon 9	c.1014C>A	p.H338Q	This study
P7	Nonsense	Exon 7	c.676C>T	p.R226X	This study
P8	Splicing error	Intron 3	c.253-1G>A	skip exon 4 ?	This study
P9	Deletion	Exons 6–8	c.484-?_897+?del	exon 6_8del	This study
P10	Missense	Exon 6	c.626A>G	p.H209R	This study
P11	Missense	Exon 7	c.731G>A	p.C244Y	This study
P12	Missense	Exon 6	c.626A>G	p.H209R	This study
P13	Deletion	Exon 10	c.1314delG	p.I439SfsX62	This study
P14	Splicing error	Intron 8	c.898-1G>A	skip exon 9 ?	This study
P15	Missense	Exon 5	c.376T>C #	p.C126R	This study
P16	Missense	Exon 12	c.1499A>G	p.D500G	This study
P17	Missense	Exon 11	c.1414G>A #	p.G472S	This study
P18	Missense	Exon 5	c.389G>C	p.R130P	This study
P19	Deletion	Exon 6	c.616delT #	p.W206GfsX7	This study
P20	Nonsense	Exon 11	c.1328G>A #	p.W443X	This study
P21	Splicing error	Intron 3	c.252+1G>C	skip exon 3 ?	This study
P22	Splicing error	Exon 11	c.1315A>G #	skip exon 11 ?	This study
P23	Deletion	Exon 10	c.1313_1314 delAGinsT	p.K438IfsX63	This study
P24	Deletion	Exon 3	c.218delG #	p.R73QfsX34	This study
P25	Missense	Exon 12	c.1498G>A	p.D500N	This study
P26	Nonsense	Exon 7	c.676C>T	p.R226X	This study
P27	Nonsense	Exon 11	c.1437C>A	p.Y479X	This study
P28	Missense	Exon 9	c.911C>G #	p.P304R	This study
P29	Nonsense	Exon 8	c.868C>T	p.R290X	This study
P30	Missense	Exon 3	c.184T>A #	p.F62I	This study
P31	Splicing error	Intron 5	c.483+1G>A	skip exon 5 ?	This study
P32	Splicing error	Intron 8, exon 9	c.[898-2_902 AGGTGGTdel] #	skip exon 9 ?	This study
P33	Deletion	Exon 3	c.185_186delTC #	p.F62X	This study
P34	Missense	Exon 6	c.613T>A	p.F205I	This study
P35	Deletion	Exons 1–13	c. (1-?_1710+?)del	exon 1–13_del	This study
P36	Missense	Exon 6	c.577T>C	p.S193P	This study
P49	Missense	Exon 1	c.1A>G	p.M1V	[21]
P50	Splicing error	Intron 1	c. 46-2A>G	skip exon 2 ?	[21]
P51	Splicing error	Intron 1	c. 46-2A>G	skip exon 2	[15]
P52	Splicing error	Exon 2	c.46_92del 47 bp c.91C>A	p.L16RfsX2	[15]
P53	Deletion	Exon 2	c. c.77_78delTT	p.F26CfsX7	[16]
P54	Deletion	Exon 2	c.91_92delCG	p.R31GfsX2	[17]
P55	Missense	Exon 3	c.162G>C	p.R54S	[15]
P56	Splicing error	Exon 3	c.252G>A	skip exon 3	[22]
P57	Splicing error	Exon 3	c.252G>A	skip exon 3	[22]
P58	Splicing error	Intron 3	c.252+2dupT	skip exon 3 ?	[22]
P59	Splicing error	Intron 3	c.252+2dupT	skip exon 3 ?	[16]
P60	Splicing error	Intron 3	c.252+5G>A	skip exon 3 ?	[16]
P61	Splicing error	Intron 3	c.252+5G>A	skip exon 3 ?	[16]
P62	Splicing error	Intron 3	c. 252+5G>A	skip exon 3	[22]
P63	Splicing error	Intron 3	c. 252+5G>A	skip exon 3	[22]
P64	Splicing error	Intron 3	c.253-3A>G	p.C85SfsX24	[16]
P65	Splicing error	Intron 3	c.253-3A>G	p.C85SfsX24	[22]
P66	Splicing error	Intron 4	c.337+1G>A	skip exon 4	[22]
P67	Deletion	Exon 5	c.345_346delCA	p.T116HfsX5	[16]
P68	Nonsense	Exon 5	c.370G>T	p.E124X	[16]
P69	Nonsense	Exon 5	c.388C>T	p.R130X	[16]
P70	Splicing error	Exon 5	c.483-484ins115 bp c.[338_483del146 bp; 484_674del191 bp]	p.K161VfsX12 p.A113QfsX2	[15]
P71	Nonsense	Exon 5	c. 469C>T	p.R157X	[15]
P72	Splicing error	Intron 5	c.483+1G>A	skip exon 5	[15]
P73	Splicing error	Intron 5	c.483+1G>C	skip exon 5	[15]
P74	Splicing error	Intron 5	c.483+1delG	p.A113DfsX16	[15]
P75	Deletion	Exon 6	c.565_568delATTA	p.I189SfsX23	[21]
P76	Deletion	Exon 6	c.565_568delATTA	p.I189SfsX23	[15]
P77	Missense	Exon 6	c.577T>C	p.S193P	[15]
P78	Nonsense	Exon 6	c.603C>G	p.Y201X	[22]
P79	Missense	Exon 6	c.626A>G	p.H209R	[22]
P80	Missense	Exon 6	c.665A>G	p.H222R	[16]
P81	Splicing error	Exons 5, 6	c.338_674del; c.484_674del	p.A113DfsX16 p.N162Tfsx14	[15]
P82	Splicing error	Intron 6	c.484_804del321 bp	p.N162_M268del	[15]
P83	Splicing error	Intron 6	c.674+6T>C	skip exon 6 ?	[21]
P84	Splicing error	Intron 6	c.674+1336 T>G; c.675-676ins81 bp	p.R226YfsX18	[15]
P85	Nonsense	Exon 7	c.676C>T	p.R226X	[15]
P86	Nonsense	Exon 7	c.676C>T	p.R226X	[15]
P87	Nonsense	Exon 7	c.676C>T	p.R226X	[16]
P88	Nonsense	Exon 7	c.676C>T	p.R226X	[16]
P89	Nonsense	Exon 7	c.676C>T	p.R226X	[22]
P90	Nonsense	Exon 7	c.676C>T	p.R226X	[22]
P91	Deletion	Exon 7	c.725_726delCA	p.T242SfsX2	[15]
P92	Splicing error	Intron 7	c.805-1G>T	skip exon 8 ?	[15]
P93	Nonsense	Exon 8	c.868C>T	p. R290X	[15]
P94	Deletion	Exon 8	c.871_881del	p.S291GfsX52	[17]
P95	Splicing error	Intron 8	c.898-1G>A	skip exon 9 ?	[25]
P96	Missense	Exon 9	c.935T>A	p.M312K	[31]
P97	Deletion	Exon 9	c.965delG	p.G322DfsX20	[15]
P98	Missense	Exon 9	c.965G>A	p.G322E	[22]
P99	Missense	Exon 9	c.1012C>T	p.H338Y	[22]
P100	Missense	Exon 9	c.1016C>A	p.P339H	[22]
P101	Deletion	Exon 9	c.1078delG	p.D360TfsX25	[15]
P102	Deletion	Exon 9	c.1078delG	p.D360TfsX25	[15]
P103	Missense	Exon 9	c.1081T>C	p.W361R	[22]
P104	Missense	Exon 9	c.1082G>T	p. W361L	[15]
P105	Missense	Exon 9	c.1082G>T	p. W361L	[16]
P106	Deletion	Exon 9	c.1095delG	p.F366SfsX19	[22]
P107	Nonsense	Exon 9	c.1120C>T	p. Q374X	[15]
P108	Deletion	Exon 9	c.1123delG	p.E375SfsX10	[15]
P109	Deletion	Exon 9	c.1123delG	p.E375SfsX10	[15]
P110	Splicing error	Exon 9/intron 9	c.1150_1151+2delAAGT	skip exon 9 ?	[16]
P111	Splicing error	Exon 9/intron 9	c.1150_1151+2 delAAGT	skip exon 9 ?	[22]
P112	Splicing error	Exon 10	c.1152G>C	skip exon 9 ? p.K384N	[16]
P113	Deletion	Exon 10	c.1170delC	p.F391LfsX13	[15]
P114	Deletion	Exon 10	c.1177delA	p.T393LfsX12	[16]
P115	Deletion	Exon 10	c.1177delA	p.T393LfsX12	[22]
P116	Missense	Exon 10	c.1234G>A	p.G412R	[22]
P117	Missense	Exon 10	c.1234 G>A	p.G412R	[21]
P118	Missense	Exon 10	c.1235G>A	p.G412E	[22]
P119	Splicing error	Intron 10	c.1151+2dupT	skip exon 10	[15]
P120	Splicing error	Intron 10	c.1315-2A>C	skip exon 11?	[15]
P121	Splicing error	Intron 10	c.1315-2A>C	skip exon 11?	[22]
P122	Nonsense	Exon 11	c.1320C>A	p.Y440X	[16]
P123	Nonsense	Exon 11	c.1320C>G	p.Y440X	[22]
P124	Deletion	Exon 11	c.1327delT	p.W443GfsX58	[15]
P125	Missense	Exon 11	c.1333T>C	p.C445R	[22]
P126	Missense	Exon 11	c.1366G>A	p.D456N	[16]
P127	Nonsense	Exon 11	c.1437C>A	p.Y479X	[22]
P128	Nonsense	Exon 11	c.1437C>A	p.Y479X	[22]
P129	Missense	Exon 12	c.1548G>C	p.W516C	[15]
P130	Missense	Exon 13	c. 1702G>A	p.E568K	[21]
P131	Deletion	Exons 7–11	c.674+608_1587-1047delinsAG	exon7_11del	[25]
P132	Deletion	Exons 1–13	c. (1-?_1710+?)del	exon 1_13del	[15]
P133	Deletion	Exons 1–13	c. (1-?_1710+?)del	exon 1_13del	[21]
P134	Deletion	Exons 1–13	c. (1-?_1710+?)del	exon 1_13del	[24]
P135	Deletion	Exons 1–13	c. (1-?_1710+?)del	exon 1_13del	[24]

Pts: patients; Nt.: nucleotide; AA.: amino acid; del: deletion; ins: insertion; dup: duplication.

#: novel mutations identified in this study.

Note: 15 patients with CYBB mutations were not included in this table because of lacking detailed mutation information from the published paper. P1–36 were patients reported in the present study and P49–135 were the patients reported previously from Mainland China.

P101 and 102 are brothers, and P108 and 109 are twin brothers [15]; while the other patients cited (except patients cited from [16]) have no relationships. The relationship between the patients who were cited from [16] could not be determined because of lacking reliable information from the published paper.

**Table 5:** Summary of different gene mutations in AR-CGD patients from Mainland China.

Patient number	Mutation gene	Mutation type	Nt. change	AA. change	Reference
P37	*CYBA*	Nonsense	c.7C>T	p.Q3X	This study
P41	*NCF1*	Deletion; missense	c.[541delG; 923T>C]★	p.D181TfsX5 p.V308A	This study
P43	*NCF2*	Missense	c.550C>T	p.R184X	This study
p136	*NCF2*	Missense	c.137T>G	p.M46R	[16]
p137	*NCF2*	Deletion	c.1130_1135delACATGG	p.D377_M378del	[16]
p138	*CYBA*	Nonsense	c.7C>T	p.Q3X	[16]
p139	*CYBA*	Missense	c.7C>T	p.Q3X	[23]
p140	*CYBA*	Missense; splicing error	c.[7C>T; 59-2A>G]	p.Q3X exon 2_del?	[23]
p141	*CYBA*	Missense	c.152T>G	p.L51R	[15]
p142	*CYBA*	Deletion	c.246_273del	p.F83SfsX98	[15]
p143	*NCF1*	Deletion	c.75_76delGT	p.Y26HfsX25	[26]
p144	*NCF1*	Deletion	c.75-76delGT	p.Y26HfsX25	[15]
p145	*NCF1*	Deletion	c.75-76delGT	p.Y26HfsX25	[15]
p146	*NCF1*	Deletion; missense	c.763-800del; c. 923T>C	p.E254_R267del p.V308A	[15]

Pts: patients; Nt.: nucleotide; AA.: amino acid; del: deletion; AR: autosomal recessive.

★: novel mutation found in this study.

P37, P41, and P43 were the patients reported in the present study and P136–146 were the patients reported previously from Mainland China.

**Table 6:** Comparison of different subtypes of CGD in 4 major PID centers from Mainland China and large cohort studies from other countries.

Pts	XL-CGD	AR-CGD			UD	Total
	*CYBB*	*CYBA*	*NCF1*	*NCF2*		
This study	36/48 (75%)	1/48 (2%)	1/48 (2%)	1/48 (2%)	9/48 (19%)	48
Center 2 [15]	30/38 (79%)	2/48 (5%)	3/48 (8%)	0/48 (0%)	3/48 (8%)	38
Center 3 [16, 20]	31/48 (65%)	1/48 (2%)	2/48 (4%)	3/48 (6%)	11/48 (23%)	48
Center 4 [21–30]	36/42 (86%)	2/42 (5%)	1/42 (2%)	0/42 (0%)	3/42 (7%)	42
Japan^*∗*^ [12]	109/148 (74%)	16/148 (11%)	10/148 (7%)	13/148 (9%)	—	229
The United States [10]	259/368 (70%)	7/368 (2%)	45/368 (12%)	10/368 (3%)	28/368 (8%)	368
Europe [11]	290/429 (68%)	22/429 (5%)	69/429 (16 %)	11/429 (3%)	37/429 (9%)	429
Turkey [32]	34/89 (38%)	20/89 (23%)	17/89 (19%)	13/89 (15%)	5/89 (6%)	89

Pts: patients; XL: X-linked; AR: autosomal recessive; UD: unidentified.

^*∗*^A total of 229 CGD patients were reported in a Japanese national registry, 148 of whom have been classified into four types based on flow cytometric and western blotting analysis.
